# Global landscape of registered clinical trials of stem cell therapy for spinal cord injury: a cross-sectional analysis

**DOI:** 10.3389/fneur.2026.1839981

**Published:** 2026-06-19

**Authors:** Yuanrun Wei, Haixin Wei, Wenhua Gong, Xuexiao Ma, Yan Wang

**Affiliations:** The Affiliated Hospital of Qingdao University, Qingdao, China

**Keywords:** clinical trials, mesenchymal stem cells, neural stem cells, regenerative medicine, spinal cord injury, stem cell therapy, translational medicine

## Abstract

Stem cell therapy is a promising regenerative strategy for spinal cord injury (SCI), but its clinical development remains heterogeneous. This study analyzed the global landscape of registered interventional trials to characterize current translational progress. We performed a cross-sectional analysis of interventional clinical trials of stem cell therapy for SCI using the Informa Trialtrove database. Records available through December 31, 2025 were screened. Observational studies, duplicate registrations, and studies not directly evaluating stem cell-based treatment for SCI were excluded. Data on trial year, country, phase, status, sponsor, cell type, and primary endpoints were extracted. Eighty interventional trials were included. Trial activity increased overall from 2008 onward, with continued study initiation in 2024 and 2025. China contributed the largest number of trials (*n* = 20), followed by the United States (*n* = 13), Spain (*n* = 10), Japan (*n* = 10), and India (*n* = 7). Most studies were early- or mid-phase, whereas phase III or later trials were rare. Completed studies accounted for 57.5%, and terminated studies for 10.0%. Academic institutions were the leading sponsors. Mesenchymal stem cells were the predominant platform, while neural lineage and induced pluripotent stem cell-derived products were emerging. Clinical development of stem cell therapy for SCI remains concentrated in early-stage trials and will require stronger efficacy evidence, endpoint harmonization, product standardization, and improved patient stratification.

## Introduction

Spinal cord injury (SCI) is one of the most devastating traumatic disorders of the central nervous system, frequently leading to persistent motor, sensory, and autonomic dysfunction and imposing a substantial long-term socioeconomic burden. Despite continued advances in acute decompression, spinal stabilization, and standardized rehabilitation, therapies capable of truly promoting neural repair and functional restoration remain extremely limited (1). In this setting, stem cell therapy has emerged as one of the most representative approaches in SCI regenerative medicine because it may exert therapeutic effects through multiple mechanisms, including cell replacement, paracrine trophic support, immunomodulation, pro-angiogenic activity, and enhancement of host neuroplasticity (2).

Preclinical animal models have played an indispensable role in advancing stem cell-based therapies for SCI, providing essential evidence on cell survival, migration, differentiation, and functional recovery prior to clinical translation. A range of SCI animal models, including rodent contusion, compression, and hemisection paradigms as well as large-animal studies, have enabled systematic evaluation of therapeutic candidates under controlled conditions, and recent reviews have comprehensively summarized the mechanisms and translational progress of these approaches (3). More broadly, animal models remain a cornerstone of surgical and implant-related research, underpinning the methodological rigor required for successful clinical development (4, 5). Beyond the field of SCI, stem cell therapy has demonstrated therapeutic potential across a wide spectrum of diseases, ranging from neurodegenerative and cardiovascular disorders to infectious conditions such as rabies, reflecting the versatility and continued expansion of this therapeutic modality (6). In parallel, artificial intelligence (AI) has emerged as a transformative tool with growing relevance to SCI research and regenerative medicine. Recent studies have explored both the potential and the limitations of AI in anatomical analysis, particularly in the context of diagnostic imaging and image-based assessment, which hold direct implications for evaluating SCI pathology and therapeutic response (7, 8). The integration of AI-based approaches may support more precise patient stratification, improved endpoint evaluation, and enhanced predictive modeling in future clinical trials of stem cell therapy for SCI.

However, the clinical translation of stem cell therapy for SCI has been far from linear: substantial heterogeneity exists with respect to cell source, route of administration, injury phase, and endpoint selection, and although early studies have repeatedly reported signals of safety and feasibility, robust confirmatory evidence sufficient to support a standard therapeutic paradigm remains scarce (2, 9). A systematic analysis of the global clinical trial landscape is therefore important for clarifying current developmental patterns, assessing the maturity of the evidence base, and informing future trial design. On this basis, the present study conducted a multidimensional descriptive analysis of clinical trials investigating stem cell therapy for SCI.

## Materials and methods

Clinical trials of stem cell therapy for SCI were identified through the Informa Trialtrove database. Previous methodological studies have shown that Trialtrove integrates registration and disclosure information from multiple sources, including ClinicalTrials.gov, and offers additional value over single registries in key variables such as trial country, study center, and trial status; moreover, the database has been widely used in high-level landscape analyses, including studies published in JAMA Network Open, to characterize research portfolios and development trends (10, 11). The search strategy was defined as [(Disease is Spinal Cord Injury) OR (Patient Segment is Spinal Cord Injury) OR (MeSH Term is Spinal Cord Injury)] AND (Drug Type is Biological > Cellular > Cell type > Stem cell), with the search period extending through December 31, 2025. Observational studies, duplicate registrations, and cell therapy studies not directly related to SCI treatment were excluded. After independent double-blind screening and cross-checking, 80 clinical trials were ultimately included. Extracted variables comprised geographical distribution, initiation year, clinical phase, trial status, sponsor type, product name, cell category, and primary study endpoints. Data were analyzed using Excel and R version 4.4.1, and visualizations were generated using Adobe Illustrator to describe the global research landscape and its temporal evolution. As this study was based exclusively on publicly accessible databases, ethics approval was not required.

## Results

**Figure 1:**
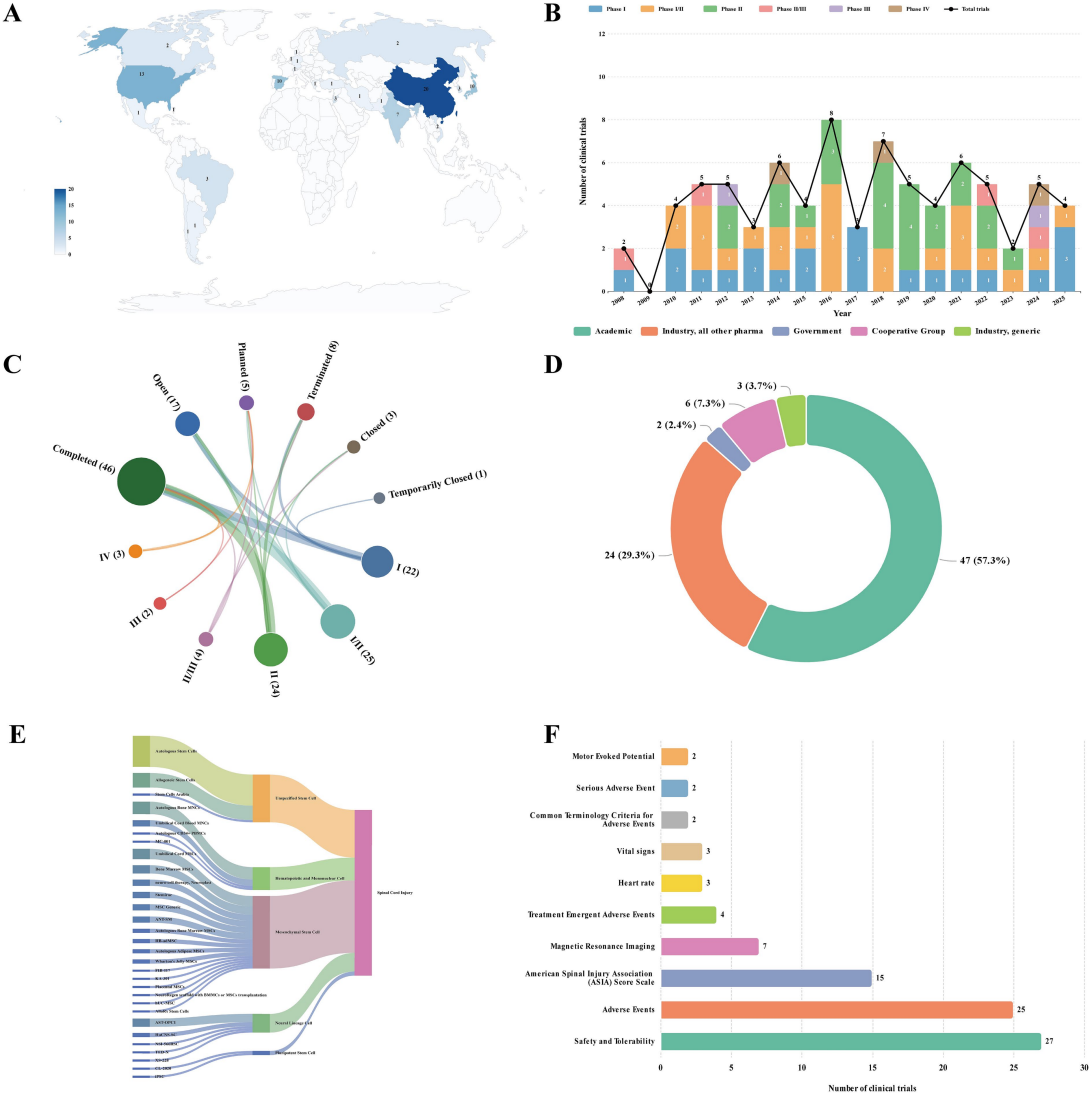
Landscape of global clinical trials of stem cell therapy for spinal cord injury. **(A)** Distribution of countries or regions included in the trials. **(B)** Temporal trends in the number of clinical trials and their phase distribution. **(C)** Cross-distribution of trial status and clinical phase. **(D)** Distribution of sponsor types. **(E)** Correlation between standardized drug names and stem cell types. **(F)** Distribution of primary endpoint types.

A total of 80 interventional clinical trials of stem cell therapy for SCI were included. Geographical analysis showed a relatively clear international multicenter pattern (Figure 1A). China accounted for the largest number of trials (*n* = 20), followed by the United States (*n* = 13), Spain (*n* = 10), Japan (*n* = 10), and India (*n* = 7). When all geographic registration entries were counted, the trials involved 24 countries or regions and 89 attribution records, suggesting that a proportion of studies had cross-regional deployment. Overall, Asia and North America represented the principal development hubs, whereas several European countries remained active in studies of specific cell types and mechanistic approaches. Temporally, the earliest identified trial dated back to 2008, after which the annual number of studies showed a fluctuating upward trend (Figure 1B). The first peak occurred in 2016, with 8 trials, and activity remained relatively high in 2018, with 7 trials. Although no marked expansion was observed after 2021, new studies continued to emerge, with 5 and 4 trials recorded in 2024 and 2025, respectively, indicating that the field has moved from an early expansion phase into one of sustained development. In terms of clinical phase, phase I/II trials accounted for 31.2% (25/80), phase II trials for 30.0% (24/80), and phase I trials for 27.5% (22/80), whereas phase III and later studies represented only 6.3% (5/80), indicating that current development remains dominated by early-stage safety validation and preliminary efficacy exploration, while confirmatory studies remain limited.

Trial status is summarized in Figure 1C. Among all included studies, 46 trials (57.5%) had been completed, 17 (21.2%) were open for recruitment or ongoing, 8 (10.0%) had been terminated, and 5 (6.2%) were planned, while the remainder were closed or temporarily closed. Completed studies were concentrated mainly in phase I, phase I/II, and phase II, indicating that a meaningful body of early clinical experience has already accumulated. At the same time, terminated trials also clustered in early-to-mid development, implying persistent challenges related to dose design, patient selection, manufacturing scale-up, and funding continuity. Analysis of sponsor type showed that academic institutions were dominant, accounting for 47 trials, followed by non-leading pharmaceutical companies with 24 trials; collaborative groups, governmental agencies, and generic pharmaceutical enterprises were involved much less frequently (Figure 1D). Taken together, these findings suggest that current clinical development in this field continues to rely predominantly on investigator-initiated studies and regionally driven innovation, with limited deep engagement from major industrial capital.

The distribution of product names and cell categories is presented in Figure 1E and Table 1. Mesenchymal stem cells (MSCs) remained the predominant technological platform, accounting for 35 entries, clearly exceeding unspecified stem cell products (23 entries), hematopoietic and mononuclear cell products (11 entries), and neural lineage cells (9 entries). Among individual products, autologous stem cells were the most common (15 trials), followed by allogeneic stem cells (7 trials), autologous bone marrow mononuclear cells (6 trials), umbilical cord-derived MSCs (5 trials), bone marrow-derived MSCs (4 trials), and AST-OPC1 (4 trials). In addition, products such as Neuro-Cells, Stemirac, HuCNS-SC, Wharton’s jelly MSCs, and iPSC-derived motor neuron progenitor cells had also entered the clinical landscape, suggesting a gradual shift from broad-spectrum cell infusion toward more engineered and lineage-directed products. With regard to primary endpoints (Figure 1F), safety and tolerability remained the central focus, with “Safety and Tolerability” appearing 27 times and “Adverse Events” 25 times; serious adverse events, treatment-emergent adverse events, and CTCAE grading were also repeatedly reported, indicating that defining the safety boundary of stem cell-based interventions remains a major priority. Efficacy endpoints were most commonly represented by the ASIA score (15 occurrences), followed by magnetic resonance imaging (MRI) (7 occurrences) and neurophysiological measures, suggesting that the evaluation framework in this field has gradually evolved toward a multidimensional system integrating safety, neurological function, imaging, and electrophysiological assessment.

**Table 1:** Standardized drug categories, names and their frequency distribution in stem cell therapy for spinal cord injury.

Category	Standardized drug names	Number of clinical trials
Unspecified stem cell	Autologous stem cells	15
	Allogeneic stem cells	7
	Stem cells Arabia	1
Pluripotent stem cell	CL-2020	1
	iPSC	1
Neural lineage cell	AST-OPC1	4
	XS-228	1
	HuCNS-SC	2
	NSI-566RSC	1
	TED-N	1
Mesenchymal stem cell	Umbilical cord MSCs	5
	Bone marrow MSCs	4
	Neuro-cell therapy, neuroplast	3
	Stemirac	3
	MSC generic	3
	ANT-SM	3
	Autologous bone marrow MSCs	2
	HB-adMSC	2
	Autologous Adipose MSCs	2
	Wharton’s Jelly MSCs	2
	FIB-117	1
	KA-301	1
	Placental MSCs	1
	NeuroRegen scaffold with BMMCs or MSCs transplantation	1
	hUC-MSC	1
	AlloRx stem cells	1
Hematopoietic and mononuclear cell	Autologous bone MNCs	6
	Umbilical cord blood MNCs	3
	Autologous CD34 + PBMCs	1
	MC-001	1

## Discussion

This analysis indicates that the global clinical trial landscape of stem cell therapy for SCI has evolved from an early, fragmented exploratory phase into a more structured stage characterized by multicenter development and the parallel advancement of several technological routes; however, its defining features remain the predominance of early-phase trials, the relative maturity of the MSC platform, the accelerated entry of neural lineage products, and the continued lack of robust confirmatory evidence. The dominance of MSCs is likely related to their relative accessibility, comparatively mature manufacturing pathways, lower immunogenicity, and broader accumulated safety experience. Nevertheless, from a mechanistic perspective, MSC-based approaches are more closely associated with microenvironmental modulation and paracrine repair than with genuine reconstruction of disrupted neural circuitry. By contrast, neural stem/progenitor cells, oligodendrocyte progenitor cells, and iPSC-derived neural lineage cells, although more demanding in terms of manufacturing complexity, tumorigenicity control, and long-term follow-up, are conceptually more consistent with the goal of structural repair in SCI, which likely explains the continued interest in programs such as AST-OPC1, NSI-566, and iPSC-derived neural progenitors (2, 12–15).

A review of trials with disclosed Trial Results suggests that the most consistent evidence available at present concerns overall safety and feasibility rather than unequivocal efficacy. For example, a phase I/II study of repeated intrathecal administration of allogeneic umbilical cord-derived MSCs supported safety and suggested preliminary functional improvement, while a 2024 pilot study of autologous bone marrow-derived MSCs in chronic complete cervical SCI showed good tolerability and no severe or moderate adverse reactions, although the observed benefit was largely limited to modest sensory improvement (9). Similarly, the AST-OPC1/LCTOPC1 program represents a relatively advanced level of standardized development for neural lineage cell therapy, as prior studies have suggested acceptable safety in subacute cervical SCI and long-term follow-up has further strengthened the safety profile of pluripotent stem cell-derived products in this setting (12, 13). These findings indicate that the central translational challenge has shifted from whether cells can be delivered safely to whether they can generate stable, reproducible, and clinically meaningful functional benefit across heterogeneous patient populations and injury settings.

Importantly, the next stage of progress is likely to depend on a transition from single-cell administration toward combinatorial regenerative strategies. Recent studies and reviews suggest that biomaterial scaffolds, injectable hydrogels, micro−/nano-delivery systems, and genetic engineering approaches may improve transplanted-cell survival, homing, axonal guidance, and synaptic integration, while artificial intelligence may support patient stratification and endpoint prediction (14, 15). This trend is also consistent with the evolving regulatory environment, including the 2025 FDA draft guidance on expedited programs for regenerative medicine therapies for serious conditions and the 2023 Chinese CDE technical guideline for clinical trials of human stem cell and derived cell therapy products, both of which emphasize early regulatory communication, product quality, and risk-based evaluation. In SCI, therefore, future development will increasingly be judged not only by biological plausibility but also by manufacturability, standardization, reproducibility, and translational feasibility within the broader framework of living drugs.

Several limitations of the present study should be acknowledged. First, the observed geographic distribution of trials may partly reflect differences in national regulatory and registration policies rather than true research activity. For instance, China’s leading position (*n* = 20) may be influenced by mandatory clinical trial registration requirements and streamlined regulatory pathways for stem cell-based products, whereas underreporting or delayed registration in other countries could lead to underestimation of their actual research output. Such regulatory heterogeneity should be considered when interpreting cross-national comparisons. Second, although Trialtrove integrates data from multiple registries including ClinicalTrials.gov and offers advantages over single-registry analyses, its coverage may not be fully comprehensive. In particular, clinical trials from non-English-speaking countries may be incompletely indexed, and some registered trials may not have been captured in the Trialtrove database. These factors may introduce selection bias and limit the generalizability of the findings. Future studies would benefit from cross-referencing multiple complementary databases and incorporating regional-language registries to achieve more complete coverage.

## Conclusion

In conclusion, the global clinical development of stem cell therapy for SCI remains centered on early- and mid-phase trials, with MSC-based products representing the most mature platform and neural lineage as well as iPSC-derived products emerging as important next-generation candidates. Although safety evidence is accumulating, high-level efficacy evidence remains insufficient. Future progress will depend less on increasing trial numbers alone than on improving patient stratification, endpoint harmonization, product standardization, and integration with enabling technologies.

## Data Availability

The original contributions presented in the study are included in the article/Supplementary material, further inquiries can be directed to the corresponding author.
